# Materia Medica, Pharmacy, Pharmacology and Therapeutics

**Published:** 1902-03

**Authors:** 


MATERIA MEDICA, PHARMACY, PHARMACOLOGY AND THERAPEUTICS. By W. HALE WHITE, M. D., F. R. C. P., Physician to and Lecturer on Medicine at Guy's Hospital, London; Author of A Text-Book of General Therapeutics. Edited by Reynold W. Wilcox, M. A., M. D., LL. D., Professor of Medicine and Therapeutics at the New York Post-Graduate Medical School and Attending Physician to the Hospital; Visiting Physician to St. Mark's Hospital; President of the American Therapeutic Society; Fellow of the American Academy of Medicine, etc. Fifth American edition, thoroughly revised. Philadelphia: P. Blakiston's Son & Co. 1901. [Price, $3.00 net.]

The editor has thoroughly revised this well-known work on materia medica bringing it abreast of the times, and thus making it one of the best works on the subject in the English tongue. The care and discretion necessary to incorporate useful unofficinal preparations and to exclude all those savoring of professionalism has been wisely exercised, and although preparations by the score have been placed on the market, but few have found their way in this work. Many chapters have been condensed and partially rewritten, with editorial notes inserted in brackets whenever found necessary.

This work can be cordially recommended both to student and practitioner and will be certain to fulfill all expectations.

Press work and binding are similar to former editions.

